# The Impact of Wrist Percooling on Physiological and Perceptual Responses during a Running Time Trial Performance in the Heat

**DOI:** 10.3390/ijerph17207559

**Published:** 2020-10-17

**Authors:** Kelsey Denby, Ronald Caruso, Emily Schlicht, Stephen J. Ives

**Affiliations:** Department of Health and Human Physiological Sciences, Skidmore College, Saratoga Springs, NY 12866, USA; kdenby@skidmore.edu (K.D.); rcaruso@skidmore.edu (R.C.); eschlich@skidmore.edu (E.S.)

**Keywords:** exercise, cooling, recovery, fatigue, thermal, environment, endurance

## Abstract

Environmental heat stress poses significant physiological challenge and impairs exercise performance. We investigated the impact of wrist percooling on running performance and physiological and perceptual responses in the heat. In a counterbalanced design, 13 trained males (33 ± 9 years, 15 ± 7% body fat, and maximal oxygen consumption, VO_2_max 59 ± 5 mL/kg/min) completed three 10 km running time trials (27 °C, 60% relative humidity) while wearing two cooling bands: (1) both bands were off (off/off), (2) one band on (off/on), (3) both bands on (on/on). Heart rate (HR), HR variability (HRV), mean arterial pressure (MAP), core temperature (T_CO_), thermal sensation (TS), and fatigue (VAS) were recorded at baseline and recovery, while running speed (RS) and rating of perceived exertion (RPE) were collected during the 10 km. Wrist cooling had no effect (*p* > 0.05) at rest, except modestly increased HR (3–5 ∆beats/min, *p* < 0.05). Wrist percooling increased (*p* < 0.05) RS (0.25 ∆mi/h) and HR (5 ∆beats/min), but not T_CO_ (∆ 0.3 °C), RPE, or TS. Given incomplete trials, the distance achieved at 16 min was not different between conditions (off/off 1.96 ± 0.16 vs. off/on 1.98 ± 0.19 vs. on/on 1.99 ± 0.24 miles, *p* = 0.490). During recovery HRV, MAP, or fatigue were unaffected (*p* > 0.05). We demonstrate that wrist percooling elicited a faster running speed, though this coincides with increased HR; although, interestingly, sensations of effort and thermal comfort were unaffected, despite the faster speed and higher HR.

## 1. Introduction

Environmental stress, specifically heat stress, increases demand placed on the cardiovascular system [1,2]. Exercise also induces stress on the cardiovascular system, and the combination of heat stress with exercise can lead to a physiological challenge where demands for blood flow begin to challenge the maximal output of the heart, eventually leading to fatigue, exhaustion, and/or a decline in performance [1,2,3,4,5,6,7].

Accordingly, researchers have been developing strategies to prevent heat stress associated declines in exercise performance. One such approach has been the use of precooling, or reducing body temperature prior to exercise in the heat [4,8,9]. A review suggested that precooling via cold water immersion likely benefits performance, where ingestion of crushed ice/water ice slurry does not likely benefit performance [4]. Although the benefits of precooling, such as with cold water immersion, are not to be ignored, the issue of practicality raises concern over feasibility of implementation, and thus alternative methods ought to be explored. Strategies of attempting to cool during exercise, termed percooling, have demonstrated a positive effect on exercise performance, on par with precooling [8,9], though studies of percooling are far less abundant.

Recently, a company has developed a wearable, active cooling method (dhamaSPORT^tm^, DhamaUSA, Scotts Valley, CA, USA) that is light weight (115 g, 6 cm wide) and can be worn on the wrist during activity while posing minimal disruption or burden to the athlete (e.g., ice vest). While we have demonstrated that this wrist cooling device improved physiological recovery and reduced fatigue from an occupationally relevant model of exercise-induced heat stress [10], it has yet to be determined whether wrist percooling is capable of improving endurance performance in the heat, and if this might impact post-exercise recovery. Aside from the obvious potential to provide cooling, mitigating exercise-induced elevations in core temperature, surface percooling might activate the transient receptor potential melastatin 8 (TRPM8) “cold receptor”, which might alter thermal sensation and/or exercise performance [11]. Further, recent work by Phillips et al. [7] suggests that cooling can modulate prefrontal cortex activation, perceptions of muscle fatigue or effort, and partially mitigate declines in local muscle performance. However, the impact of wrist percooling on perceptual responses during exercise in hypothermia is unknown.

Therefore, the purpose of this study was to investigate whether the wrist cooling improves exercise performance in the heat or lessen physiological strain, and if this effect is “dose-dependent.” We hypothesized that wrist percooling would reduce perceptions of effort and thermal stress, reduce heart rate, and/or improve performance on a 10 km running time trial, and these effects would be greater with the active cooling of both wrists. Second, use of the cooling bands would improve recovery as assessed by heart rate, heart rate variability, core temperature, and reduce fatigue and thermal sensations, all of which would also be greater with the active cooling of both wrists.

## 2. Materials and Methods

### 2.1. Subjects

**Figure 1:**
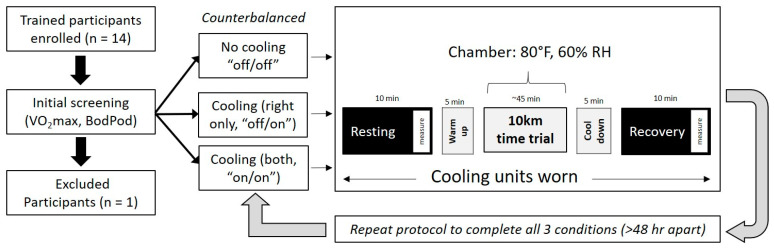
Experimental overview: RMR, resting metabolic rate; IET, incremental exercise test.

Fourteen exercise-trained healthy male volunteers between the ages of 18 and 54 years were recruited for this study (Figure 1). To participate in this study, all participants must have been regularly exercise training for more than one hour at least three times a week for the past four months, have a maximal oxygen consumption (VO_2_max) of >45 mL/kg/min, and >1 year of experience in competing in running events (e.g., 5 km, 10 km, half-, full-, ultra-marathon, half-, full-ironman, etc.). Participants were screened, via health history, and those with cardiovascular, pulmonary, musculoskeletal, or metabolic disease, those taking regular medication, or presenting contraindications to the ingestible telemetry pill (*n* = 1) were excluded. Methodologically, women were excluded to avoid the long periods of time that would be necessary (up to 3 months) to ensure adequate recovery with parallel desire for testing to occur in a singular phase of the menstrual cycle, thus reducing the impact of hormonal fluctuations. All participants provided written informed consent prior to any testing. The protocol was approved by the Institutional Review Board of Skidmore College (IRB # 1612-568) and was conducted in accordance with the most recent revisions to the Declaration of Helsinki.

### 2.2. Study Overview

The current study was conducted in a single blind, counterbalanced, crossover design to investigate the potential impact of wrist cooling on performance in, and recovery from, exercise in the heat (Figure 1). As the number of participants did not equal or equally multiply by the number of possible sequences of three trials, we used a Latin square approach to counterbalance. All testing was conducted in the Environmental Physiology Laboratory at Skidmore College. For each visit, participants were asked to avoid strenuous exercise for 24 h prior and alcohol/caffeine use 12 h prior to each study visit. Participants were instructed to maintain a similar diet and sleep regimen throughout the duration of the study. Participants were asked to wear shorts and t-shirt and to dress similarly across trials. Finally, participants were instructed to arrive each day fueled and hydrated as if preparing for a race, which included drinking the proper amount of fluids prior to each experimental visit (e.g., ~500 mL 2–3 h prior and 250 mL within 15 min of the visit). All participants reported to the laboratory on four separate occasions: a screening day and three experimental trials. While wearing two cooling bands, the three trials were conducted as follows: (1) both bands were off (off/off), (2) one band on (off/on), (3) both bands on (on/on). In the off/on condition, the right wrist was always activated. All experimental trials for a subject were completed at the same time of day to reduce impact of diurnal variation. In a thermoneutral (21 ± 1 °C, 29 ± 12% relative humidity) and normobaric (~750 mmHg) environment, the first screening visit assessed participant characteristics, which included anthropometrics (height, weight), body composition using air displacement plethysmography (BodPod, Cosmed, Chicago, IL, USA), and aerobic fitness via graded exercise testing on a treadmill (PPS Med, Woodway, Waukesha, WI, USA). A running protocol (modified McConnell) [12,13] was used to determine maximal oxygen consumption (VO_2_max) using open circuit spirometry and gas analysis (TrueOne 2400, Parvomedics, Sandy, UT, USA) [14]. Prior to each experimental trial, participants were given an FDA approved core temperature telemetry pill (HQ Inc, Palmetto, FL, USA), which was taken 8–12 h prior to the study visit [15,16].

### 2.3. Procedures

**Figure 2:**
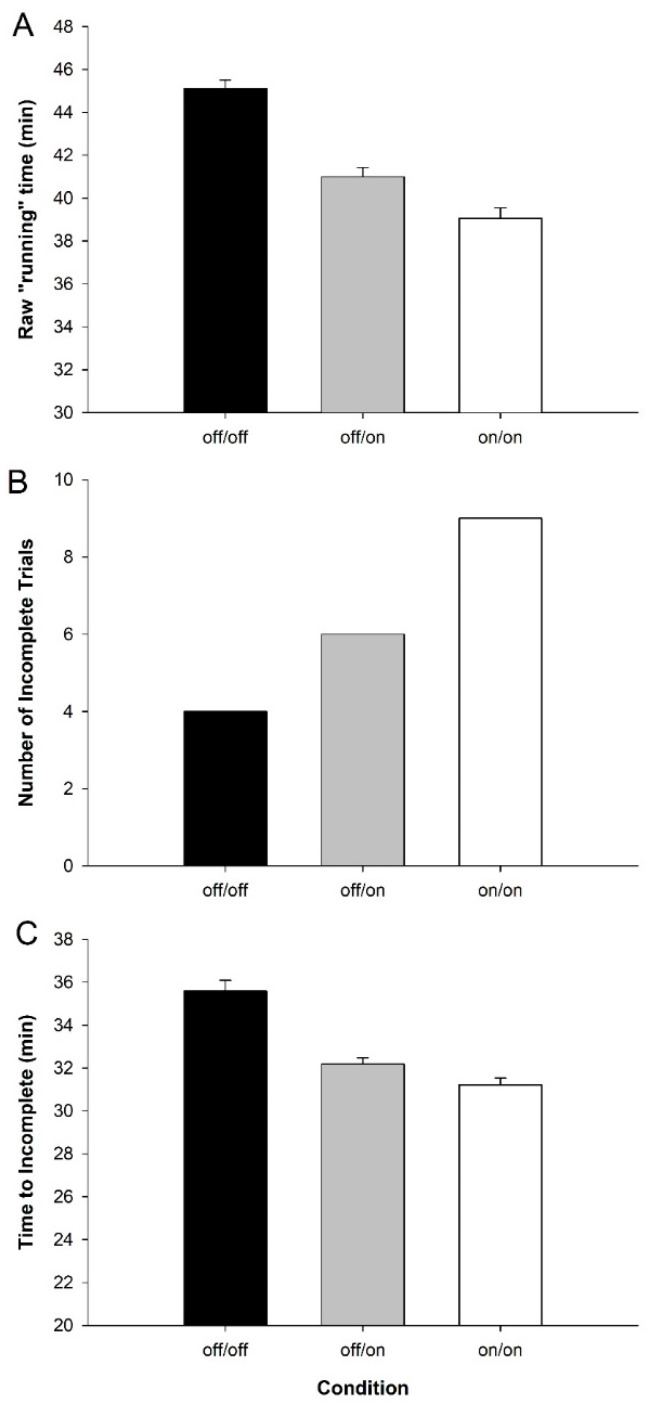
Raw performance data for all trials (*n* = 13): (**A**) average run time, including incomplete trials across condition; (**B**) number of incomplete trials across condition; and (**C**) average time to incompletion across conditions. Data are means ± Standard Error.

Upon arrival, a urine sample was collected and hydration status was confirmed via urine specific gravity (USG < 1.020) as described previously [17]. If USG was >1.020, participants were given 500 mL of water and USG was retested thereafter (though as the participants were familiar with race preparations, this only occurred once out of 39 total visits). Participants were then instrumented with a heart rate monitor (H7, Polar USA, Lake Success, NY, USA), and the presence of the core temperature telemetry pill was confirmed (CorTemp Recorder, HQ Inc, Palmetto, FL, USA). Participants were then seated and were outfitted with two wrist cooling bands (Dhama Sport Pro, Dhama USA, Scott’s Valley, USA) (Figure 2). In the “on” condition, the bands were activated and set to the coolest setting (7.2 °C). While we attempted to avoid investigator cues and reduce possible anticipatory responses by single blinding and not making the participants’ aware of which condition they were receiving, when the band was active, participants’ were able to detect the cooling, but when the band was off (off/off condition) they were unsure. The device elicits cooling through a one-inch square ceramic cooling plate placed over the anterior vascular portion of the wrist, which dissipates heat via Peltier effect over a larger heat sink area on the exterior portion of the device. The heat transfer rate for this device ranges from 0.2 to 200 watts, with typical values of 0.5–50 watts, depending upon ambient conditions. After 10 min of quiet rest, a one minute [18], breathing frequency paced [19], recording of heart rate (HR) and R-R intervals were obtained via HR monitor, sent to a mobile device (IPad Pro, Apple, Cupertino, CA, USA) via Bluetooth™ and analyzed by a mobile device application (Elite HRV, Gloucester, MA, USA). The Elite HRV application performs artifact correction and has been shown to be valid [20] and has been used in previous studies [10,20,21,22]. Specifically, along with mean HR, R-R intervals were analyzed for the standard deviation of R-R intervals, SDNN; root mean square of successive differences, RMSSD; and the log transformed RMSSD, LnRMSSD. HRV was measured to assess potential impacts of wrist cooling on recovery as it is an increasingly recognized method to assess or monitor athlete acute and chronic physiological response to training, or recovery and readiness to train [22,23,24,25,26]. After HR and HRV were obtained, to further characterize potential impacts of wrist cooling on recovery, blood pressure (BP) was measured via oscillometric cuff method (Mobilograph, GmbH, Stolberg, Germany) [27,28,29], after which thermal sensation/comfort via thermal sensation (TS) scale (0 “unbearably cold” to 8 “unbearably hot”), and fatigue via a visual analog scale (0 “no fatigue” to 10 “severe fatigue”) were recorded.

Participants were then allowed to warm up for a maximum of 5 min outside the chamber in the thermoneutral laboratory, typically followed by use of the restroom to void their bladder. Subsequently, participants entered the heated environmental chamber (26.7 °C, 60% relative humidity, heat index of 28 °C, “caution”) and were instructed to complete the 10 km time trial (~6.2 miles, since the treadmill was in English units) as fast as possible at 0% grade. Thus, participants were able to see their speed and allowed direct control of the treadmill speed. Verbal encouragement was provided to all participants in a consistent manner between subjects and across trials. Participants were allowed to drink water, ad libitum, during all trials, but were asked to consume fluid in a similar volume and manner across trials, matched for their first trial completed. During exercise, participants were asked to report their thermal sensation, rating perceived exertion using standardized visual scales every 5 min, while HR and core temperature (T_CO_) were monitored continuously and recorded every minute. Due to safety concerns, and institutional restrictions, in effort to avoid heat related injury, if core temperature reached two consecutive measures of 39.1 °C, or a single measure of 39.2 °C or higher, the trial was ended and the participant was immediately removed from the chamber and into a cool-down period in the thermoneutral laboratory. In such case, post-measures were obtained in an identical manner as if they had completed the trial.

Once the 10 km trial was completed, participants were escorted from the chamber and completed a 5 min cool-down, walking on a treadmill in the thermoneutral laboratory. HR and T_CO_ were continuously monitored for safety reasons. Fifteen minutes after the cessation of the exercise, a post-exercise assessment of the baseline measures, except USG, was performed, namely: VAS, thermal sensation, core temperature, HR, HRV, and BP. The timing of the post-exercise measurements was maintained for all trials, including those that were ended due to core temperature reaching our institutional safety threshold. Once post-exercise measures were obtained, the wrist cooling units were turned off and removed. Participants reported back to the laboratory to complete the other two trials in a randomized counterbalanced order as described above. Visits were completed with a minimum of 48 h in between (average time between visits ~72 h).

### 2.4. Statistical Analysis

Data were analyzed using commercially available software (SPSS v26, IBM, Armonk, NY, USA) As the number of athletes who reached our institutional safety cutoff turned out to be larger than anticipated, additional analyses were conducted to compare the number of incomplete trials between wrist percooling conditions using a chi square test, and pairwise comparisons were used to determine if the time to incompletion differed between conditions. Further, a Kaplan–Meier survival curve analysis was conducted to compare the rate and time of incompletion using a log rank test. Again, due to athletes’ core temperatures reaching our institutional safety cutoff, to allow direct time-matched pairwise comparisons between trials, all trial data were analyzed to the point at which we had complete data for all participants (16 min), as well as the final data point for each participant. The final data point was either the final data at incompletion due to reaching the temperature cutoff or the final value at the end of the 10 km time trial. Thus, data were analyzed and plotted to the longest common time, plus each athlete’s final data point, and only for one athlete in one condition were 16 min and final the same. Further, to estimate effects of wrist percooling on 10 km time trial performance, if the trial was incomplete for reaching core temperature cutoff (see Figure 2), trial performance was estimated using average running speed for the trial.

Prior to analysis, any anomalous individual data points presenting as an outlier (>2 SD) were removed from the data set, and where appropriate, interpolated using a linear function. Accordingly, heart rate, core temperature, and speed were analyzed using a 3 (condition) by 17 (time, 16 min + final) repeated measures ANOVA. For RPE and TS, a 3 (condition) by 4 (time) repeated measures ANOVA was completed. To compare the pre- and post-measurements, a 3 (condition) by 2 (time) repeated measures ANOVA was completed for HR, core temperature, RMSSD, MAP, SDNN, LnRMSSD, diastolic blood pressure (DBP), systolic blood pressure (SBP), VAS, RPE, and TS Significance was established at *p* < 0.05. Data are presented as means ± standard deviation (SD), unless indicated otherwise.

## 3. Results

### 3.1. Participant Characteristics

The participant characteristics are presented in Table 1. Most participants (*n* = 10 of 13) were active triathletes, having competed in half or full distance Ironman events as well as running events, but all had road and/or trail running racing experience. Participants were fit, with an average VO_2_ max of 59 mL/kg/min (range 50–71), particularly considering their average age of 33 years.

### 3.2. Effects of Wrist Cooling on Baseline Parameters

No significant differences were found in resting core temperature, indicators of heart rate variability, blood pressure, thermal sensation, rating of perceived exertion, or in reported fatigue using a visual analog scale between conditions (Table 2). However, use of the bands tended to affect heart rate (*p* = 0.05), where HR was elevated by ~5 beats/min in the off/on condition (Table 2).

### 3.3. Ten km Time Trial Performance

**Figure 3:**
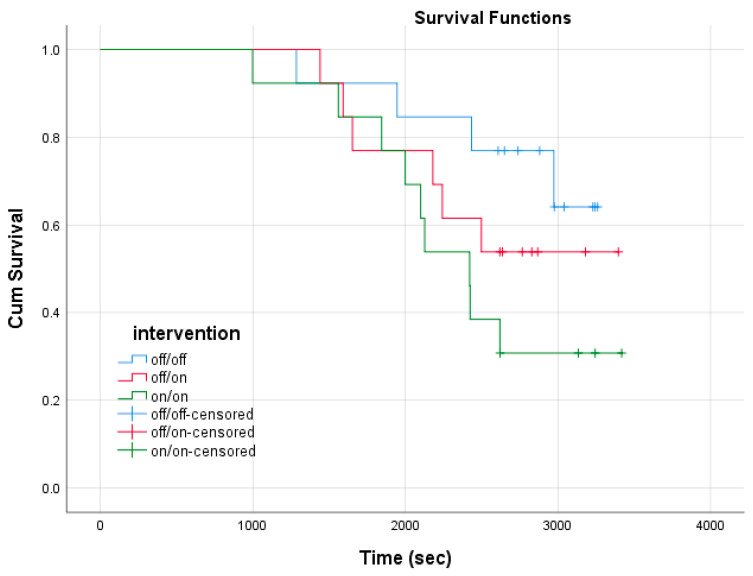
Kaplan–Meier survival curve across time (seconds) between wrist cooling conditions during the 10 km time trial.

Raw running time, including any incomplete trials, tended to decrease with the use of the bands (Figure 2A, off/on *p* = 0.14 and on/on *p* = 0.08 vs. off/off). However, due to participants reaching our institutionally mandated core temperature safety cut off, and importantly not of volitional means, this aforementioned time is tainted by a number of incomplete trials that tended to increase with the use of the bands (Figure 2B), and trended to an earlier incompletion time (Figure 2C). Chi squared analysis found that the proportion of incomplete trials did not significantly differ by condition (*p* = 0.16), nor were times to incompletion also not different between trials (pairwise comparisons, *p* = 0.51–0.81). Additionally, Kaplan–Meier survival curve analysis using a log rank test revealed no significant differences in survival distribution between wrist percooling conditions (*p* = 0.14, Figure 3).

**Figure 4:**
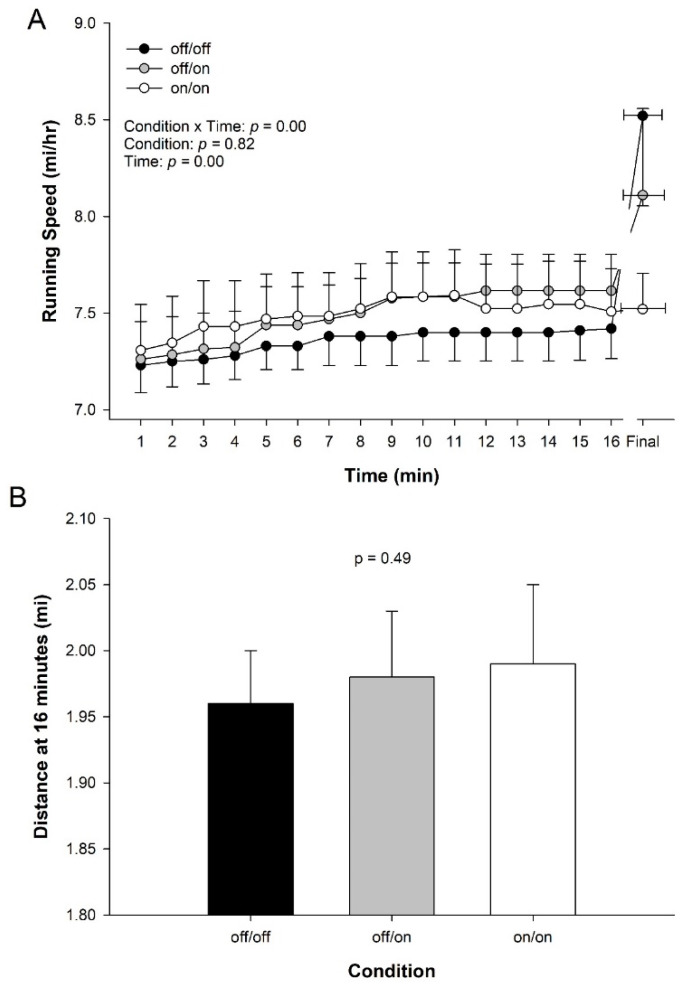
Running performance across wrist percooling condition. (**A**) Self-selected running speed during the 10 km time trial in the heat. Results of two-way ANOVA are presented (inset). Note: due to safety tolerance in core temperature trials were ended early and plotted to the shortest time, plus each athlete’s final data point (with SE for time). (**B**) Distance to 16 min across wrist percooling condition (*n* = 13). This time was chosen as it was the longest point to which all participants completed at least 16 min for all 3 trials. Data are means ± Standard Error.

There was a significant interaction of wrist percooling condition and time on running speed, where RS tended to increase more over time with wrist percooling (Figure 4A), though there was no main effect for condition (*p* = 0.13). Focusing on the time to which all participants had completed, the distance achieved at 16 min was not different between conditions (off/off 1.96 ± 0.16 vs. off/on 1.98 ± 0.19 vs. on/on 1.99 ± 0.24 miles, *p* = 0.490, Figure 4B). Using both actual or projected 10 km times, there was no statistically significant effect of the bands (off/on *p* = 0.49 on/on *p* = 0.77 vs. off/off), despite a tendency for an approximate 30 s improvement in 10 km time for the off/on condition and a 10 s improvement in 10 km time in the on/on condition (off/off: 50:14.6, off/on: 49:45.9, on/on: 50:04.2 min:s). Using only those with complete trials, within a condition, the trend is less clear (off/off: 49:17.4, off/on: 50:02.3, on/on: 48:51.3 min:s).

### 3.4. Physiological Response to 10 km Time Trial in the Heat

**Figure 5:**
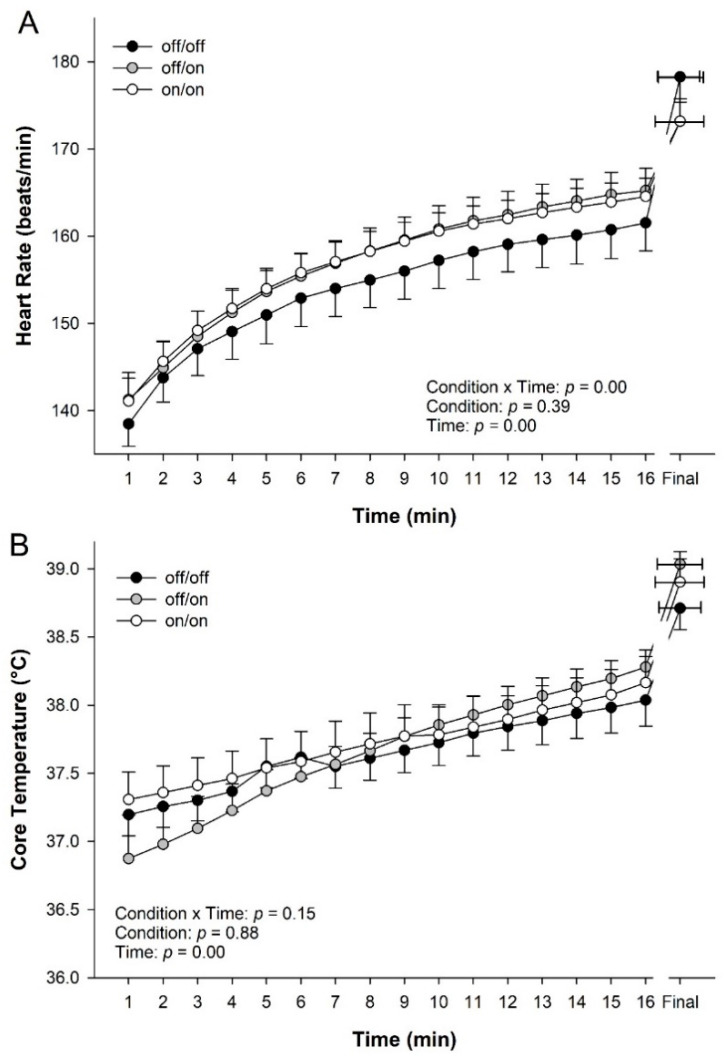
Physiological responses to 10 km time trial in the heat across wrist percooling condition. (**A**) Heart rate and (**B**) core temperature during 10 km time trial. Note: due to safety tolerance in core temperature, trials were ended early and plotted to the shortest time, plus each athlete’s final data point. Data are means ± Standard Error (*n* = 13).

A significant interaction between condition and time was found for heart rate (*p* = 0.00, Figure 5A). Expectedly, a main effect for time was found for heart rate throughout the trial (*p* = 0.00). No significant differences were found for heart rate between conditions (*p* = 0.39).

There was no significant interaction of condition by time for core temperature during the 10 km time trial (TT) (*p* = 0.15, Figure 5B). No main effect of condition was found during the 10 km time trial for core temperature (*p* = 0.88). Expectedly, however, there was a significant effect of time on core temperature during the trial (*p* < 0.001, Figure 5B).

### 3.5. Perceptual Measures during 10 Km Time Trial in the Heat

**Figure 6:**
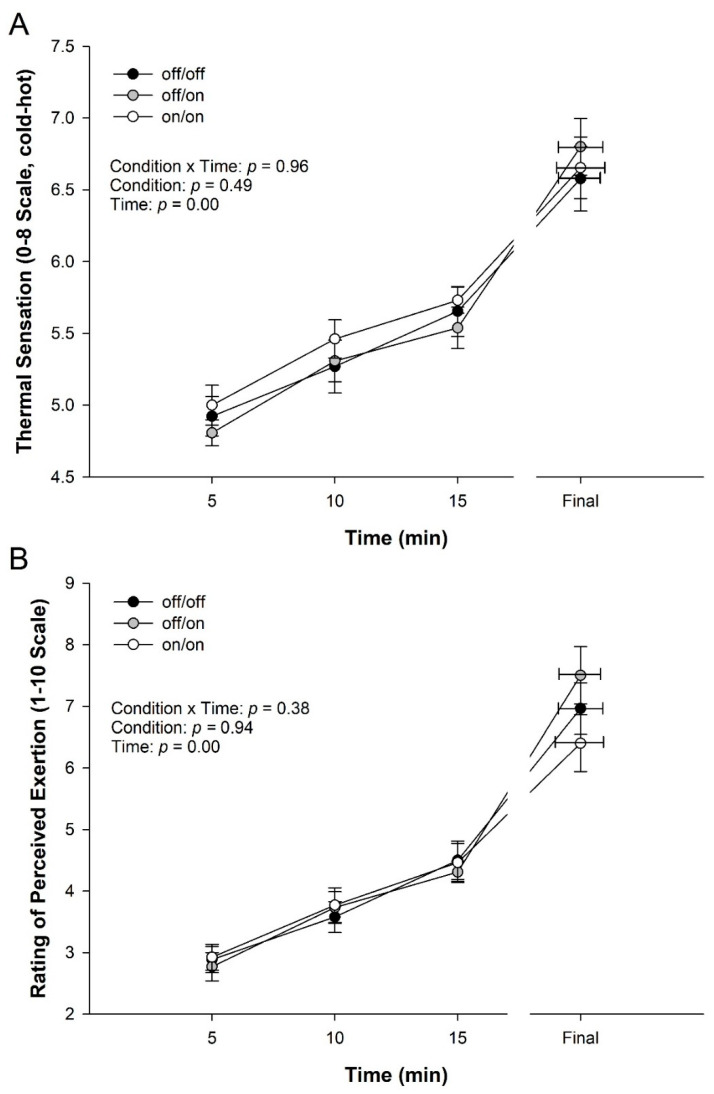
Perceptual measures during 10 km TT. (**A**) Thermal sensation (TS) and (**B**) Rating of perceived exertion. Note: due to safety tolerance in core temperature, trials were ended early and plotted to the shortest time, plus each athlete’s final data point. Data are means ± Standard Error, (*n* = 13).

There was no significant interaction between condition and time for thermal sensation during the 10 km (*p* = 0.96, Figure 6A). A significant main effect was found for time, where the participants’ TS increased over time (*p* = 0.00), though no significant differences were observed between conditions for TS (*p* = 0.47).

No significant interaction of condition and time was found for RPE (*p* = 0.38, Figure 6B). Naturally, a main effect for time was found (*p* = 0.000). However, RPE did not significantly differ between conditions (*p* = 0.93).

### 3.6. Impact of Wrist Cooling on Recovery

Pre- and post-measurements are shown in Table 2. No significant interaction (*p* = 0.58) was found between condition and time for core temperature, though core temperature was on average 0.2 to 0.7 °C cooler in recovery with use of the bands. Although core temperature approached significance, there was not a significant effect of time (*p* = 0.05); core temperature was not different from baseline and had recovered. However, there was no effect of condition on core temperature (*p* = 0.64).

There was no significant interaction between condition and time for heart rate (*p* = 0.36, Table 2). There was a significant main effect for time (*p* = 0.00) on heart rate with elevations post-exercise. Heart rate significantly differed between conditions, where heart rate tended to increase with the use of the bands (condition effect *p* = 0.03). To measure heart rate variability, the root mean square of the successive differences (RMSSD) was measured. No significant interaction effect for condition by time (*p* = 0.23) and no main effect of condition was found (*p* = 0.97). A significant main time effect was found for RMSSD (*p* = 0.000, Table 2) with a significantly reduced RMSSD post-exercise. In addition, there was no significant interaction effect for condition by time (*p* = 0.43) and no difference between conditions (*p* = 0.96) for log transformed RMSSD (LnRMSSD). Expectedly, similar to RMSSD, there was a main time effect for LnRMSSD (*p* = 0.00, Table 2) with lower HR variability post-exercise. Lastly, SDNN had no significant condition effect (*p* = 0.41) or condition by time effect (*p* = 0.15). Corresponding to the other heart rate variability variables, there was a main time effect for SDNN (*p* = 0.00) with lower HR variability post-exercise.

No significant interaction for condition by time was found for mean arterial pressure, MAP (*p* = 0.90, Table 2). During the recovery process, a main effect of time was found for MAP (*p* = 0.01) with lower MAP in recovery, though no significant differences between conditions were found (*p* = 0.08). Systolic blood pressure, SBP, showed no significance for main time effect, condition by time, or condition (all *p* > 0.05, Table 2). Diastolic blood pressure, DBP, had no significant interaction of condition by time (*p* = 0.28) or effect of condition (*p* = 0.11). In contrast to SBP, DBP had a significant main time effect (*p* = 0.02) with a reduction in diastolic pressure post-exercise.

During recovery, there was no significant interaction between condition and time for RPE (*p* = 0.08) and TS (*p* = 0.72). There was a significant main effect of time for RPE and TS (both *p* = 0.000, Table 2). No significant differences between conditions were present for RPE (*p* = 0.43) and TS (*p* = 0.33). There was no significant interaction between condition and time for the fatigue visual analog scale (*p* = 0.47, Table 2). Expectedly, a main effect of time was present during the recovery process for VAS (*p* = 0.00), showing higher reported levels of fatigue, but no significant difference was found between conditions in the recovery process (*p* = 0.10).

## 4. Discussion

The intent of this study was to ascertain whether percooling via wrist cooling bands would improve 10 km running time trial performance in the heat, or lessen the physiological strain, and enhance recovery. The main finding of this study was that the use of the bands seemed to promote the participants to run at a faster speed over time. Thus, when using the bands, there was a corresponding increase in heart rate over time as a result of this increased speed and energy demand. On average, core temperature, thermal sensation, and rating of perceived exertion were not different when using the bands. Use of the bands did not appear to alter baseline or enhance physiological or perceptual indicators of recovery from the 10 km running bout. There was also no clear evidence that two bands were more advantageous than one, in terms of the physiological and perceptual responses to exercise, performance, or recovery. Thus, athletes considering use of wrist percooling, wearing one band is likely sufficient and possibly optimal. In conclusion, the cooling bands elicited a faster running speed over time; however, this comes at a physiological cost, but surprisingly not a perceptual one. Further work in the field or in unrestricted settings are needed to ultimately demonstrate efficacy of wrist percooling.

### 4.1. Ten km Time Trial Performance

In the present study, we observed that wrist percooling through the use of wrist cooling bands seemed to elicit a faster running speed in the participants over time (interaction effect, Figure 4). The faster self-selected running speed over time could be interpreted as an increase in performance because, all else held constant, would be expected to lead to a faster 10 km time trial. Focusing on distance covered at a common time, the distance covered by the athletes was not statistically higher at 16 min, a critical time point in our study. Using completed and estimated 10 km times, wrist percooling via use of the band’s lead to times that were on average ~20 s faster, but were not significantly different from control. The faster running speed, as an indicator of performance, agrees with prior research using precooling, which also observed an increase in performance [9,30,31,32,33,34].

Previous reviews indicated that the magnitude of the effect of precooling likely depends upon the modality of precooling (e.g., water immersion, and depth, ice slurry, cooling garment, etc.) and how performance is assessed (i.e., time to exhaustion, and prescribed intensity, time trial, distance trial, etc.) [4,9]. Specifically, cold water immersion elicits an effect size (Cohens d) of 0.4 to 2 on performance vs. control, whereas precooling garments elicit an effect size of 0.1 to 0.5 (small to medium) [4], the latter more reflecting the magnitude effect in the current study (Cohens d effect size of 0.2, small effect, in off/off vs. off/on). Although the trend for a positive effect on actual or estimated 10 km time was not statistically significant, it should not be interpreted necessarily as useless. A 10–30 s effect on 10 km performance could have practical implications. To put this modest effect into perspective, when looking at the professional men’s results of the 2016 AJC Peachtree road race, one of the nation’s largest 10 km events, run in Atlanta, GA, during July (similar environmental conditions at race start to those in our study), a 20 s boost in performance could mean being on the podium or not. For the Bolder Boulder 10 km race, one of the largest 10 km races in the world, first place through sixth place in the pro division was separated by 20 s. Again, further field testing is needed to support this notion, though as climatic temperatures rise and running events are held in hot environments (e.g., Tokyo 2021 Olympic games), developing viable methods of supporting athlete’s performance is increasingly paramount.

### 4.2. Impact of Wrist Percooling on Physiological Responses to 10 km TT in the Heat

In the current study, core temperature during the 10 km time trial was unaffected by percooling via wrist cooling bands (Figure 5). The present findings are in contrast to previous work using precooling, which demonstrated reduced core temperature particularly during initial stages of exercise [31,32,35,36]. For example, in a study by Lee and Haymes [32], 30 min of precooling reduced core temperature at rest and during exercise, though final core temperatures were not different between precooled and control. In agreement, when using a mix-method of ice bags and a cooling vest, Duffield et al. [31] saw a decrease in core temperature during warm up and the first sprinting periods; however, there was no significant difference in the final core temperature. Precooling is thought to reduce core temperature, creating a larger reservoir or tolerance for core temperature increases, postponing increases in temperature to the latter stage of exercise. In the current study, it was hypothesized that the use of the bands would blunt the rise of core temperature and lower it during the 10 km time trial, but this was not observed. However, given the running speed was increased over time, further challenging the cooling capacity of the unit due to the additional metabolic heat load, in already loaded condition, observing such an effect may not be possible. The cooling power of the device, maximally 200 watts, is simply not capable of ablating human heat production, estimated at over 1000 watts [37], but whether it might attenuate the rise in core temperature in individuals warrants further study.

Concordant to the increased running speed over time seen with wrist percooling, heart rate also was elevated over time during the 10 km time trial (Figure 5). These results somewhat support one previous study done by Duffield et al. [31], where they found no significant difference in heart rate during the exercise. However, previous research found with various models of precooling that HR was suppressed [30,32,33,35,38], at least during the initial stages of exercise, as final HR often was not different between conditions. However, some of these protocols used steady state exercise models [35], others incremental [33,38], or time to exhaustion [30,32], where speed or work load were matched. Thus, the higher heart rate over the trial with the use of the bands, while perhaps in disagreement with previous studies and not supporting the hypothesis, makes physiological sense in the context of the increased running speed.

### 4.3. Impact of Wrist Percooling on Perceptual Measures during 10 km TT in the Heat

The current study found no significant difference between conditions and no interaction effect for RPE (Figure 6). In support of the present data, Duffield et al. [31,36] also found no significant difference between the precooling and control conditions for RPE during the performance trial. However, other studies showed that RPE decreased with the use of precooling methods during the exercise performance [35,39]. However, in both of these studies walking/running speed was fixed.

Similar to RPE, in the present study, thermal sensation or perception during the 10 km time trial was not different between conditions and did not see an interaction effect with the use of the bands (Figure 6). Only one previous study supports the present data that showed no significant difference between conditions during the performance [31]. Other research has proven that with the use of cooling, the TS decreases during exercise [35,39,40].

Thus, while the present data does not support the hypothesis that the use of the cooling bands would have lowered RPE and TS, the present data are to be considered in the context of altered running speed over time. Recent meta-analysis suggests that topical or ingestion of menthol, a known agonist of the “cold receptor,” TRPM8, can alter thermal sensation and/or exercise performance [11], perhaps independent of core temperature [41]. Relatedly, work by Phillips et al. [7] suggests that precooling might modulate the prefrontal cortex and/or its processing of afferent feedback regarding perceptions of effort, fatigue, or skin temperature, which might support greater exercise tolerance, and ultimately an attenuation of muscle fatigue. Thus, while it is tempting to speculate that RPE and TS might have been expected to increase in response to the increased running speed, and that the cooling bands mitigated the expected increase in perception of effort and thermal strain, further work is needed to confirm this hypothesis and explore the potential psychophysiological effects of wrist percooling.

### 4.4. Impact of Wrist Percooling on Physiological Recovery

In contrast to our initial hypothesis, core temperature did not significantly differ between conditions during recovery (Table 2). A prior meta-analytical review demonstrated that more aggressive cooling methods, such as whole body cooling likely help to recover performance [42], though few studies have focused on recovery of core temperature. Much of this work has been done in occupational models, such as firefighting, using multiple interventions, some invasive, to induce cooling and recovery of heart rate after firefighting [43,44,45,46,47]. Accordingly, our previous work using this wrist cooling device to induce cooling after an occupational model of exercise-induced heat stress via exercise in encapsulating clothing, revealed a significant positive impact of wrist cooling on recovery of temperature and heart rate [10]. However, in that study the exercise was necessarily more modest (walking) and shorter, thus the rise in core temperature was lower, all only increasing by 1 °C or less, potentially creating multiple differentials between the present and the aforementioned study. Interestingly, heart rate was found to be significantly impacted by wrist cooling at rest, and agrees with previous work that demonstrated skin cooling to 7 °C resulted in a modest 5 beat/min increase, likely the result of activating sensory afferent neurons [48]. This resting difference, we believe, contributed to a main effect of condition when exploring rest and recovery, as post-exercise HR values were not actually different between conditions. In support of no difference in heart rate during recovery between conditions, Edmonds et al. [40] using the wrist cooling device also found no significant difference in heart rate after high intensity physical activity. Thus, wrist cooling may be insufficient to hasten recovery of HR after high intensity activity in the heat.

### 4.5. Impact of Wrist Percooling on Recovery of Perceptual Measurements

During the recovery period, there was no significant difference in TS. However, TS during the recovery period was recorded highest in the off/off condition (4.6) and the lowest in the on/on condition (4.2). In support of this, Edmonds et al. [40] found a significant decrease during the recovery period, at the 10 min marker. The present data do show a lower value with the use of the bands; however, due to the time to incompletion decreasing with the bands, and the number of incomplete trials increasing, perceptual cues could be altered due to the decrease in performance. In recovery, RPE, or perhaps more appropriately the fatigue visual analog scale values after the exercise did not significantly differ, which does disagree with our prior work [10] using wrist cooling. Although for the reasons mentioned above, this may be expected. Future studies should explore the potential impact of wrist cooling on recovery in the field or athletic setting where immediate cooling applications may not be readily available and thus wrist cooling could perhaps be a bridge from or to more powerful cooling methods, such as cold-water immersion.

### 4.6. Experimental Considerations

The 10 km time trial took place in a controlled environment during the winter months and therefore the athletes were not acclimated to running in hot and humid environments. Institutional safety mandated cessation of exercise just above 39 °C (Figure 2), impairing our ability to determine whether performance would have truly been affected; indeed, previous work has suggested that high-level athletes are capable of tolerating such core temperatures or higher, perhaps even to 41.5 °C [49]. Anecdotally, none of the participants exhibited any heat-related illness signs or symptoms, suggestive of a greater tolerance, and this may have underestimated potential performance effects as the athletes were unable to fully execute their individual race plan (e.g., negative splits or sprint at the end). Nonetheless due to this cutoff, in trials where the athlete reached this threshold we estimated or projected their performance. Future studies are needed to determine possible effects in a relatively unrestricted or field environment to observe fully the potential effects on performance. Although ingestible temperature telemetry pills have been demonstrated valid and able to track changes over time with heating or cooling [15,16], there was some variability in core temperature measurements with the telemetry pill and future studies might consider using more invasive measures such as esophageal or rectal thermistors. It was impossible to conduct the study in a fully blinded or placebo-controlled manner, though the research team sought to minimize eliciting any anticipatory responses, and participants wore both bands during all three trials. Measures of skin temperatures and/or localized thermal sensations would have enhanced the study, and future studies using this cooling method should include these measures, as well as pulmonary measures (e.g., VO_2_, ventilation, respiratory exchange ratio).

## 5. Conclusions

In the present study, wrist percooling during a 10 km TT in the heat resulted in a faster self-selected running speed and higher heart rates, though thermal sensation or perceptions of effort were unaffected. The increased running speed over time with wrist percooling might be practically meaningful, but further work is needed to determine the potential impacts of wrist cooling on performance, particularly in the field.

## Figures and Tables

**Table 1:** Subject characteristics (*n* = 13).

Variable	Means ± SD
Age (years)	32.6 ± 8.9
Height (cm)	178.6 ± 9.1
Weight (kg)	76.9 ± 8.0
Body Mass Index (kg/m^2^)	24.1 ± 2.1
VO_2_Max (mL/kg/min)	59.1 ± 5.2
Body Fat %	14.8 ± 6.8
Fat Free %	80.5 ± 15.3
Body Fat Mass (kg)	11.5 ± 5.4
Body Fat Free Mass (kg)	65.4 ± 7.4

**Table 2:** Pre- and post-measurements for all three conditions (*n* = 13).

Variable		Off/Off	Off/On	On/On
HR (beats/min)	Pre	56.0 ± 7.0	61.0 ± 8.0 ^#^	59.0 ± 6.5
	Post	86.0 ± 6.0	87.0 ± 6.0	87.0 ± 6.8 *^,†^
Core Temperature (°C)	Pre	37.0 ± 0.5	37.2 ± 0.6	37.1 ± 0.7
	Post	38.0 ± 1.0	37.3 ± 1.6	37.8 ± 1.0
MAP (mmHg)	Pre	106 ± 11	109 ± 11	106 ± 10
	Post	101 ± 6	104 ± 8	99 ± 11 *
SBP (mmHg)	Pre	124 ± 13	125 ±13	120 ±12
	Post	119 ± 10	120 ± 9	113 ± 16
DBP (mmHg)	Pre	85 ± 12	85 ± 12	86 ± 10
	Post	81 ± 8	80 ± 10	80 ± 10*
LnRMSSD (a.u.)	Pre	4.6 ± 0.4	4.5 ± 0.4	4.56 ± 0.5
	Post	3.4 ± 0.5	3.5 ± 0.7	3.4 ± 0.6 *
SDNN (ms)	Pre	159.0 ± 65.4	133.8 ± 36.3	146.5 ± 45.6
	Post	59.6 ± 28.2	64.1 ± 45.1	64.5 ± 29.1 *
TS (0–8)	Pre	3.4 ± 0.6	3.2 ± 0.4	3.5 ± 0.6
	Post	4.6 ± 0.9	4.2 ± 1.2	4.5 ± 0.8*
RPE (1–10)	Pre	2.0 ±1.0	1.5 ± 0.7	2.0 ± 0.6
	Post	4.5 ± 1.6	5.0 ± 2.0	4.0 ± 2.0 *
Fatigue (VAS 1–10)	Pre	1.4 ± 1.3	1.7 ± 1.1	1.2 ± 1.0
	Post	6.2 ± 1.8	6.4 ± 1.6	5.3 ± 2.5 *

HR, heart rate; MAP, mean arterial pressure; SBP, systolic blood pressure; DBP, diastolic blood pressure; LnRMSSD, natural log transformed root mean squared of successive differences; A.U., arbitrary units; SDNN, standard deviation of normal R-R intervals; Msec, milliseconds; TS, thermal sensation; RPE, rating of perceived exertion; VAS, visual analog scale. * main effect of time; † main effect of condition, # vs. off/off, *p* < 0.05. Note: all time effects were *p* < 0.05 pre vs. post. Means ± SD.
